# Purification and Activity Testing of the Full-Length YycFGHI Proteins of *Staphylococcus aureus*

**DOI:** 10.1371/journal.pone.0030403

**Published:** 2012-01-20

**Authors:** Michael Türck, Gabriele Bierbaum

**Affiliations:** Institute of Medical Microbiology, Immunology and Parasitology (IMMIP), University of Bonn, Bonn, Germany; National Institutes of Health, United States of America

## Abstract

**Background:**

The YycFG two-component regulatory system (TCS) of *Staphylococcus aureus* represents the only essential TCS that is almost ubiquitously distributed in Gram-positive bacteria with a low G+C-content. YycG (WalK/VicK) is a sensor histidine-kinase and YycF (WalR/VicR) is the cognate response regulator. Both proteins play an important role in the biosynthesis of the cell envelope and mutations in these proteins have been involved in development of vancomycin and daptomycin resistance.

**Methodology/Principal Findings:**

Here we present high yield expression and purification of the full-length YycG and YycF proteins as well as of the auxiliary proteins YycH and YycI of *Staphylococcus aureus*. Activity tests of the YycG kinase and a mutated version, that harbours an Y306N exchange in its cytoplasmic PAS domain, in a detergent-micelle-model and a phosholipid-liposome-model showed kinase activity (autophosphorylation and phosphoryl group transfer to YycF) only in the presence of elevated concentrations of alkali salts. A direct comparison of the activity of the kinases in the liposome-model indicated a higher activity of the mutated YycG kinase. Further experiments indicated that YycG responds to fluidity changes in its microenvironment.

**Conclusions/Significance:**

The combination of high yield expression, purification and activity testing of membrane and membrane-associated proteins provides an excellent experimental basis for further protein-protein interaction studies and for identification of all signals received by the YycFGHI system.

## Introduction

**Figure 1:**
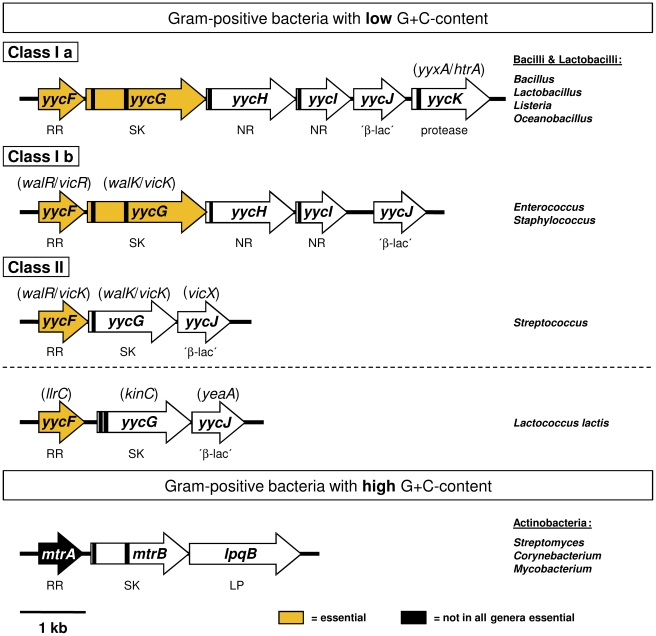
Gram-positive bacteria with orthologous *yyc* (*wal*/*vic*) genes. The upper part of the figure shows the structural organization of orthologous *yyc* operons in Gram-positive bacteria with low G+C-content adopting the classification into class I and II proposed by Szurmant et al. [5]. To distinguish between genera that contain 5 or 6 cistrons, the class I operon was divided into two subclasses. For a clear overview, orthologous genes are named according to the corresponding *yyc* gene in *Bacillus subtilis* in all genera and synonymous terms commonly used in a genus are given in brackets. The lower part of the figure illustrates the organization of the *mtrAB*/*lpqB* operon in Gram-positive Actinobacteria with high G+C-content. The operons are drawn to scale from representative species: *Bacillus subtilis* 168 (NC_000962), *Staphylococcus aureus* N315 (NC_002745), *Streptococcus pneumoniae* R6 (NC_003098), *Lactococcus lactis* Il1403 (NC_002662), *Corynebacterium glutamicum* ATCC 13032 (NC_006958). Essential genes are highlighted. TM coding regions were determined utilizing the TMHMM 2.0 server 2.0 web interface (http://www.cbs.dtu.dk/services/TMHMM/) and marked as black bars. Functions are indicated below the arrows: RR = response regulator, SK = sensor kinase, NR = negative regulator of kinase function, ‘β-lac’ = similarities to an enzyme super-family containing metallo-β-lactamases, protease = serine protease, LP = conserved lipoprotein. Within the class II of *yyc* operons, the YycG kinase of *Lactococcus lactis* is an exception of the rule, because it possesses two transmembrane domains (instead of one), which flank a very short extracytoplasmic loop comprising four amino acids [7], [8].

*Staphylococcus aureus* is a prominent agent of nosocomial disease and has shown an unusual aptitude in acquiring resistance to antimicrobial agents. A search for essential novel antibiotic targets in *S. aureus* revealed the existence of an essential two-component regulatory system (TCS) similar to the genes *yycFG* from *Bacillus subtilis* [1], [2]. The YycG kinase is generally anchored by two transmembrane (TM) sequences in the cytoplasmic membrane and possesses a periplasmatic loop of 142–147 amino acids. YycF is the cognate response regulator [3]. Further investigation in *B. subtilis* showed that the genes *yycFG* form a part of a larger operon that comprises *yycFGHIJ* [4]. Disruption of *yycH* or *yycI* led to an upregulation of a YycF regulated promoter, indicating that YycH and YycI might interact with YycG and thereby modulate YycG activity [5]–[7]. Similar systems are widespread among Firmicutes [3], [4] and also present in Actinobacteria [8] (**Fig. 1**).

The YycF regulon in *B. subtilis* comprises proteins involved in cell wall metabolism and turn-over [9], [10]. In *S. aureus*, a search using the *Bacillus* consensus YycF recognition site yielded 31 loci with a YycF binding motif [11] among them nine proteins with proven autolytic function or similarity to autolysins, which are upregulated upon induction of *yycFG* [12]. Two of these autolysins (LytM and SsaA) account for cell viability and thereby for the essentiality of the YycFG TCS [13]. Recently, it was suggested to rename YycFG (VicRK) into WalRK [12]. In the recent literature this term is increasingly used for the diverse orthologous kinases and response regulators, but only infrequently for the complete operon. For reasons of clearness, we therefore decided to retain the original *yyc* designation for this report.

Interestingly, YycFGHI also seems to influence antibiotic resistance in *S. aureus*. An intermediately vancomycin resistant clinical isolate, *S. aureus* SA137/93A, is characterized by overexpression of *yycFGHI* that was caused by insertion of an IS element, IS*256*, into the *yycFGHI* promoter region. Overexpression *in trans* employing an inducible promoter also enhanced resistance to vancomycin in *S. aureus* [14]. Furthermore, point mutations and a premature stop codon in *yycG* [15] as well as inactivation of *yycH* [16] have been associated with increased resistance to daptomycin.

Here, we describe for the first time the expression and purification of the full-length kinase YycG, and the development of *in vitro* test systems comprising the response regulator YycF, using detergent-micelles and phospholipid-liposomes. The successful reconstitution of YycG into unilamellar vesicles (ULVs) will enable the characterization of the Yyc proteins and their signal cascade in a suitable transmembrane environment that mimics the *in vivo* situation and represents a major improvement on previous systems which only characterized the cytoplasmic domains of YycG [11], [17]. Even kinetic studies on YycG of *S. aureus* and *S. pneumoniae* have been limited to truncated kinase versions so far [11], [18].

The YycG(Y306N) protein described here is a mutant of YycG that upon overexpression in *S. aureus* increased resistance to vancomycin even further [14]. In order to demonstrate the influence of the mutation, that is located in the intracellular Per-Arnt-Sim (PAS) domain of the kinase, on the activity of the protein, it was tested along with its wild type variant. The results indicate that this mutation might lead to an activation of YycG in a membrane environment as present after reconstitution of the protein into liposomes.

## Results

### Cloning and purification of the proteins of the YycFGHI system

YycG, YycH and YycI are membrane proteins. In order to facilitate the expression of the His_6_-tagged constructs in *Escherichia coli*, to enhance correct folding and to avoid the formation of inclusion bodies, the genes *groESL* were amplified from the genome of *E. coli* K12 JM109 (primers see **Table 1**) and cloned into the plasmid pREP4, thereby removing the *lacI* repressor gene. The resulting plasmid pREP4groESL(MT) was introduced *in trans* into the *E. coli* expression strains BL21(DE3), C41(DE3) and C43(DE3). The C41(DE3) and C43(DE3) host strains have been derived from strain BL21(DE3) by selection for their ability to express membrane and toxic proteins [19].

**Table 1:** Oligonucleotide primers used in this study.

Primer	Sequence (5′→3′)	Annealing Temp.	Reference
a) Oligonucleotides used for cloning of the *yyc* genes into the expression plasmid pET22bΔpelB:			
vicR-For	AAACCATGGGTATGCAAATGGCTAG (*Nco*I)*	70.1°C	this work
vicR-Rev	CGACTCGAGCTCATGTTGTTGGAGG (*Xho*I)	70.1°C	this work
vicK-For	GGTCCATGGGAATGAAGTGGCTAAA (*Nco*I)	65.5°C	this work
vicK-Rev	TCCCTCGAGTTCATCCCAATCACCG (*Xho*I)	65.5°C	this work
yycH-For	CACCCATGGGTGTGAAGTCATTGAA (*Nco*I)	70.1°C	this work
yycH-Rev	GTTCTCGAGTTCAAGCCTCCCATCG (*Xho*I)	70.1°C	this work
yycI-For	GGCCCATGGGAATGAACTGGAAACT (*Nco*I)	53.0°C	this work
yycI-Rev	TTCCTCGAGATGATTAATAATTTTA (*Xho*I)	53.0°C	this work
b) Oligonucleotides used for sequencing of pET22bΔpelB-derived plasmids:			
T7 promoter	TAATACGACTCACTATAGG	ND	Novagen
T7 terminator	GCTAGTTATTGCTCAGCG	ND	Novagen
vicK innen	GCTTTAGCATTTAATAACTTGTCTA	ND	this work
c) Oligonucleotides used for cloning of *groESL* into pREP4groESL(MT):			
groESL-For	CTTGCTAGCAGGAGAGTTATCAATGAATATTCG (*Nhe*I)	57.3°C	this work
groESL-Rev	GCAGTCGACTTACATCATGCCGCCCATG (*Sal*I)	57.3°C	this work
d) Oligonucleotides used for sequencing of *groESL* harboured on pREP4groESL(MT):			
pREP4_groESL-For	CACATATTCTGCTGACGCACCGGTG	ND	this work
pREP4_groESL-Rev	AATCCGTCGGCATCCAGGAAACCAG	ND	this work
groESL_innen	AACTGATCGCTGAAGCGATGGACAA	ND	this work

*Restriction sites are indicated in brackets and underlined in the sequence. ND = Not determined.

The genes *yycF*, *yycG*, *yycH* and *yycI* were amplified using the DNA of *S. aureus* SA137/93A as a template (primers see **Table 1**). The plasmid pET22bΔpelB, which provides a C-terminal His_6_-tag, was employed as vector for expression in *E. coli*. The *pelB*-leader sequence had been removed from this plasmid to allow intracellular expression [20].

Only the response regulator YycF-C-His_6_ and the auxiliary protein YycH-C-His_6_ could be expressed in the host strain *E. coli* BL21(DE3) pREP4groESL(MT) after induction with 1 mM IPTG and incubation for 4 h at 30°C and were successfully purified from the cell lysate employing a Ni-NTA affinity column under standard conditions (up to 10.6 and 7.7 mg protein per litre of culture). In contrast, the full-length constructs of the histidine kinase, YycG-C-His_6_, and the mutant kinase YycG(Y306N)-C-His_6_, which both harbour two transmembrane sequences, could not be detected in the standard strain *E. coli* BL21(DE3) pREP4groESL(MT) after induction with IPTG and were only expressed in sufficient amounts in *E. coli* C41(DE3) pREP4groESL(MT) or *E. coli* C43(DE3) pREP4groESL(MT). Indeed, after induction with 1 mM IPTG and subsequent incubation for 16 h at 30°C, the kinases were incorporated into the membranes of the *E. coli* cells and were purified from the membrane fraction employing up to 39 mM DDM, leading to a yield of up to 1.3 mg/l for both proteins. Since *E. coli* C43(DE3) pREP4groESL(MT) expressed higher amounts of both kinases, the auxiliary protein YycI-C-His_6_ was purified from the membrane fraction of this strain and yielded up to 6.6 mg/l of protein. The purified kinases were stored in dialysis buffer I or II containing 50% glycerol at −20°C for up to one year without loss of activity.

### Oligomeric state analysis employing blue native polyacrylamide gel electrophoresis

Blue native polyacrylamide gel electrophoresis (BN-PAGE) employs gradient gels and proteins loaded with Coomassie Brilliant Blue (CBB) to confer the necessary negative charges for migrating through the gel. In the absence of SDS, higher oligomers will persist and the protein complexes will stop migrating when the size of the protein complex is too large for the pores in the gel. For the proteins of the Yyc system, formation of oligomers was expected, since the kinase monomers autophosphorylate each other *in trans*, thus, the formation of dimers is required. According to bacterial two-hybrid studies with the *B. subtilis* proteins, YycG forms a ternary complex with the auxiliary proteins YycH and YycI and moreover all three proteins are able to form homo-dimers [6]. For YycI of *Bacillus subtilis*, the formation of a dimer had been described for the crystal structure [21]. In the case of YycH, the formation of homo- or hetero-oligomers through TM helix interaction has been suggested, but homo-dimerisation could not be confirmed by the crystalization studies, since the fragment of YycH lacked the N-terminal TM domain [22]. Nevertheless, hetero-oligomerization of the YycGHI proteins is a key mechanism of controlling the YycG kinase activity [6], [7], [23]–[25].

**Figure 2:**
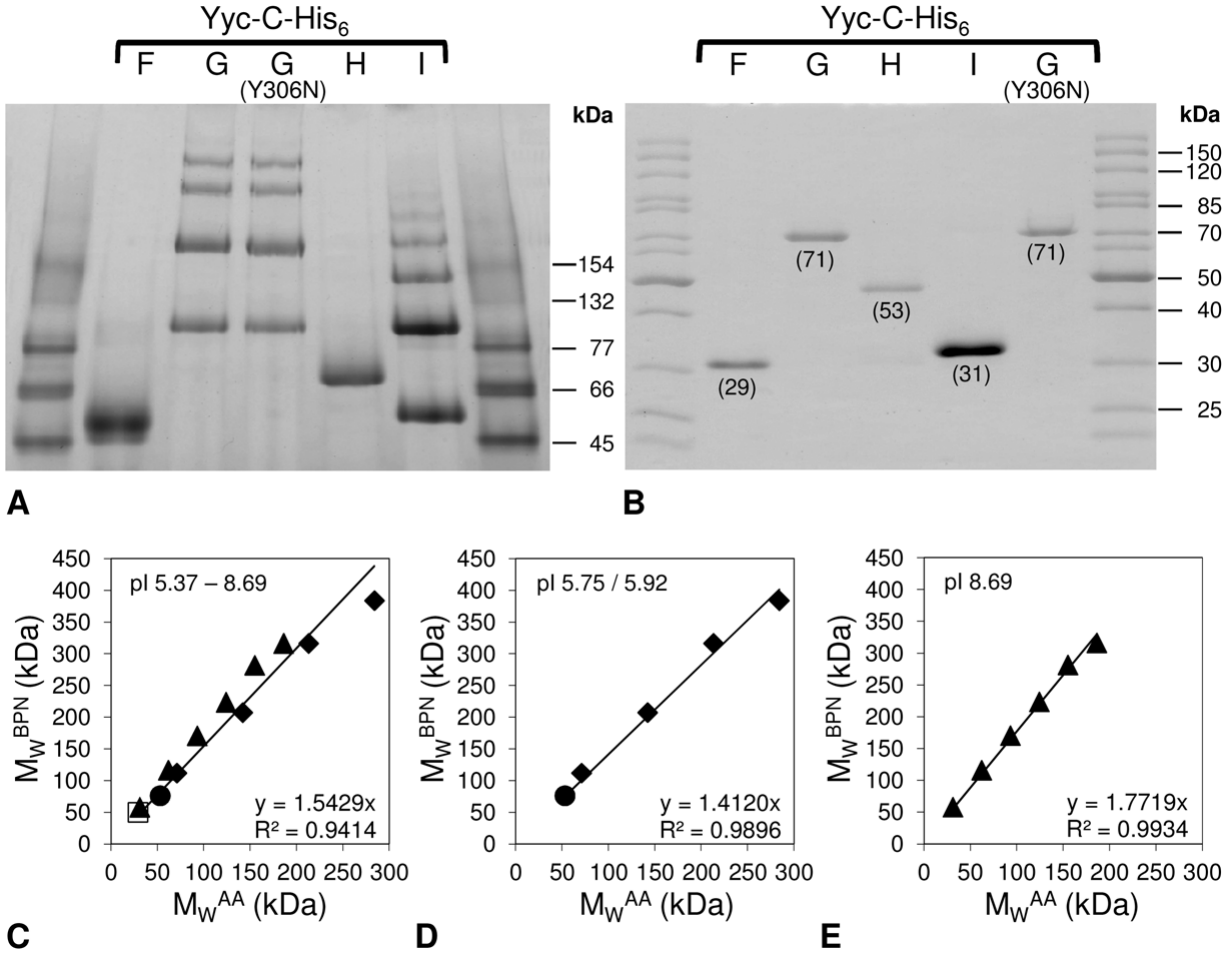
Oligomeric state analysis via BN-PAGE. (A) Multimeric organization of all C-terminal His_6_-tagged full-length Yyc proteins in this work as observed in blue native polyacrylamide gel electrophoresis (BN-PAGE). Marker proteins in BN-PAGE (kDa): Conalbumin (dimer) 154, BSA (dimer) 132, conalbumin (monomer) 77, BSA (monomer) 66 and ovalbumin 45. The molecular weights (M_W_) of the Yyc-C-His_6_ proteins on basis of their amino acid sequence are shown in brackets. As a control, the separation of all purified His_6_-tagged full-length Yyc proteins by SDS-PAGE is shown in (B). (C)–(E): The apparent discrepancy between the molecular weights and migration distances of the Yyc-C-His_6_ constructs in BN-PAGE can be solved by application of a conversion factor, which is deduced by linear regression from the correlation of the M_W_ as calculated by amino acid sequence (M_W_^AA^) and the apparent molecular weight observed in BN-PAGE (M_W_^BPN^), respectively. Symbols: YycF (open square), YycG (closed diamond), YycH (closed circle) and YycI (closed triangle).

Here, oligomeric state analysis was performed employing a 4–20% gradient polyacrylamide gel and 5 µg of protein of each of the C-terminal His_6_-tag constructs of YycF, YycH and YycI and 10 µg of YycG and YycG(Y306N). In BN-PAGE, the migration behavior of proteins varies according to their capacity to bind CBB [26] and their number of negative charges [27]. Especially when TM rich membrane proteins are compared to soluble marker proteins, a massively decreased electrophoretic mobility of the membrane proteins is often observed [28]. **Fig. 2 (A)** shows that the electrophoretic mobility of the C-His_6_-tagged Yyc proteins in the BN-PAGE was also lower than that of the soluble marker proteins, i.e. the observed molecular masses in the BN-PAGE (M_W_^BNP^), calibrated with soluble marker proteins, were higher than the calculated molecular masses (M_W_^AA^) or the masses determined in SDS-PAGE (**Fig. 2 (B)**). Since there is a linear correlation between the observed and calculated masses in BN-PAGE [28], an average conversion factor f of 1.5 (M_W_^BNP^ = f×M_W_^AA^) was determined by linear regression for all Yyc proteins as shown (**Fig. 2 (C)**) (coefficient of determination, R^2^ = 0.94). However, a higher accuracy (R^2^ = 0.98 or 0.99, respectively) was achieved, when the proteins were grouped according to their pIs and the factors were calculated separately for both groups, as shown in **Fig. 2 (D)** and **(E)**. With application of these specific conversion factors, the values for M_W_^AA^ could be calculated from the M_W_^BNP^ values within 10% error. As shown in **Fig. 2 (A)**, only one band was visible for YycF-C-His_6_ and YycH-C-His_6_, indicating that no oligomers were formed. In contrast, both kinases, YycG-C-His_6_ and YycG(Y306N)-C-His_6_, formed up to four bands, with molecular weights that indicated their ability to exist as dimers, trimers, and tetramers *in vitro*. For the auxiliary protein YycI-C-His_6_ even up to six bands were visible and the molecular weights concurred with the formation of multimers of YycI-C-His_6_. The aberrant migration behaviour of TM rich membrane proteins has previously been explained by their ability to bind high amounts of CBB with their hydrophobic transmembrane regions [28]. Since the Yyc-C-His_6_ proteins contained no (YycF), one (YycI, YycH) or two TM domains (YycG) (**Fig. 1**), respectively, this aberrance may be also due to the pI and especially the introduction of the positive charges of the His_6_-tag might play a role.

Complex formation between the different Yyc proteins was also tested, but could not be demonstrated in the BN-PAGE system; similar data were obtained using cross linking experiments with formaldehyde (data not shown). This result might be due to the fact that the different Yyc proteins interact via their membrane domains [25] which may require a more hydrophobic and membrane-like environment for interaction, which is not provided during BN-PAGE.

### *In vitro* phosphorylation assays in the detergent-micelle-model

In a first step, the autophosphorylation activity of the purified His_6_-tag constructs of the sensor kinases was tested. To this end a detergent-micelle-model (with Triton X-100 as membrane mimicking surfactant) employing a mixture of unlabeled ATP and [γ-^33^P]ATP, followed by SDS-PAGE and autoradiography was established. All reactions were started by addition of the ATP mixture.

In first experiments, the buffer system that had been employed previously for the activity tests of the N-terminally truncated, soluble version of the YycG kinase of *S. aureus* (‘YycG) [11] was also used for the full-length YycG variants, but the proteins failed to demonstrate their activity. Only after very prolonged incubation times (>60 min) and excessive exposition of X-ray films weak, phosphorylated bands of lower molecular weight than expected for the kinases appeared on the autoradiogram, indicating that, under these conditions, fragmentation of the kinases had occurred. Furthermore, in this buffer, addition of different concentrations of MgCl_2_ did not show any effect on the activity.

In the next step, the reaction buffer was supplemented with diverse detergents to create more suitable conditions for the full-length kinases still possessing transmembrane domains. Thus, a weak kinase activity was observed in the presence of Triton X-100 and CHAPS, but almost no activity at all could be detected in the presence of the other tested detergents (CHAPSO, deoxyBIGCHAP, N-laurylsarcosine, DDM).

**Figure 3:**
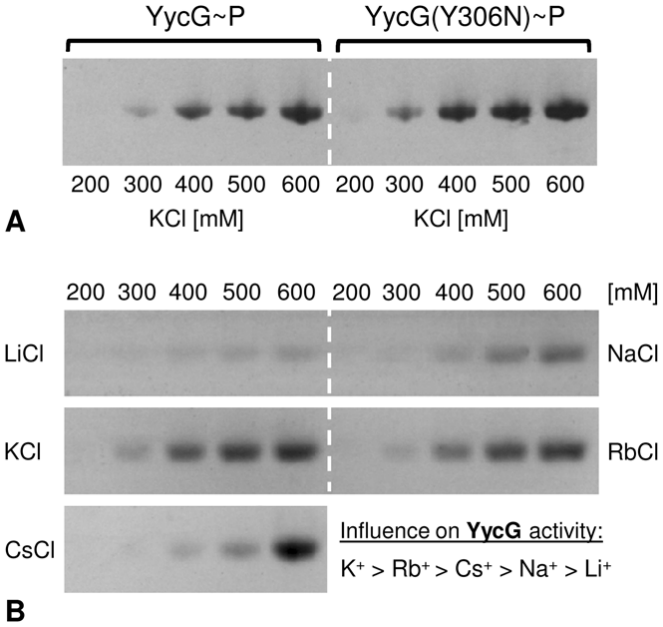
Autophosphorylation activity in the detergent-micelle-model. (A) Activity of the YycG and the YycG(Y306N) kinase after incubation for 60 min at RT in presence of 8.5 mM Triton X-100, 25 µM ATP, 4 µCi [γ-^33^P]ATP and various KCl concentrations. (B) Activity of YycG in presence of diverse alkali salts in various concentrations under the same conditions.

**Figure 4:**
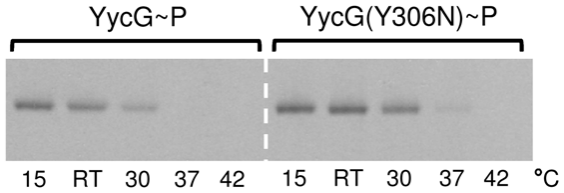
Influence of temperature/fluidity in the detergent-micelle-model. Activity of the YycG and the YycG(Y306N) kinases after incubation for 60 min in presence of 8.5 mM Triton X-100, 25 µM ATP, 4 µCi [γ-^33^P]ATP and 500 mM KCl at different temperatures to induce changes in microviscosity of the membrane mimicking surfactant Triton X-100.

In order to analyse the influence of increased monovalent cation concentrations on phosphorylation, the effect of addition of 200–600 mM KCl to the sample buffer was tested. **Fig. 3 (A)** shows that the optimal activity of the full-length protein was reached only in presence of 500 to 600 mM KCl. Furthermore, a comparison with the molecular weight size markers in the SDS-PAGE showed distinct phosphorylated bands at ∼70 kDa without any observable fragmentation, as calculated for the full-length proteins. **Fig. 3 (B)** shows that an increase in the concentrations of other alkali salts (LiCl, NaCl, RbCl and CsCl) enhanced the activity as well. The efficacy of the tested ions decreased in the following order: K^+^>Rb^+^>Cs^+^>Na^+^>Li^+^, and since Rb^+^ and Cs^+^ exerted stronger effects than Na^+^, the ion radii seemed to be of greater impact than the physiological relevance of the ions. Therefore, a specific effect of K^+^ on the activity of YycG was unlikely, especially as relatively high concentrations had to be present. More likely the increased level of cations, especially of K^+^, had an influence on the microenvironmental conditions, i.e. the Triton X-100 micelles. Indeed, the presence of alkali salts increases micellar hydration, aggregation number and thereby also size of Triton X-100 micelles [29], [30]. From these results, it had been initially assumed that an increased micellar hydration might lead to faster solvation dynamics of the water molecules entrapped within the micellular palisade player [31], but in a later work actually a reverse trend was observed, indicating that along with increasing concentrations of monovalent salts also the microviscosity inside the Triton X-100 palisade layer increased [32]. This finding was explained by the assumption that alkali cations, residing in the palisade layer, increased hydration, but that, moreover, their own hydration inside the palisade layer led to a clustering of water molecules and thereby increased the microviscosity [32]. At lower concentrations an exception of this rule seemed to account for the behaviour of Li^+^ ions which are able to bind to the ether groups inside of the palisade layer. Here, addition of LiCl led to a hydration of Triton X-100 micelles and a concomitant decrease of microviscosity until all free ether groups had been saturated. Beyond this concentration a sudden increase in microviscosity of the micelles was observed, caused by the hydratisation of the excess of surplus unbound Li^+^ ions [33]. This hypothesis could explain why LiCl was found to exert almost no influence on YycG activity. Taken together, the modulation of the activity of the micelle bound YycG kinase by different alkali ions might reflect a direct response of the kinase to changes in fluidity in its microenvironment. In order to confirm this hypothesis, the effect of different temperatures on the activity of YycG was tested, since changes in temperature have a striking influence on hydration and microviscosity of Triton X-100 micelles [31], [34]. **Fig. 4** shows that an increase of the temperature above RT led to a decrease in the activity of YycG and interestingly, its activity was highest at the lowest temperature (15°C). This result was unexpected since *S. aureus* grows best between 30°C and 37°C. However, if the activity of the kinase were to be directly dependent on the fluidity of its microenvironment, it might be concluded from these results, that a certain degree of compactness of Triton X-100 micelles must be given to stimulate YycG. This would also explain why under elevated temperatures the kinase was less active, since it can be assumed that fluidity increases with temperature in general. In comparison, the activity of the mutant YycG(Y306N) kinase version seemed to be less affected by variations in temperature. Since a high activity for YycG was demonstrated in presence of 500 mM KCl at RT, all further experiments were performed at these conditions unless indicated otherwise in the text.

**Figure 5:**
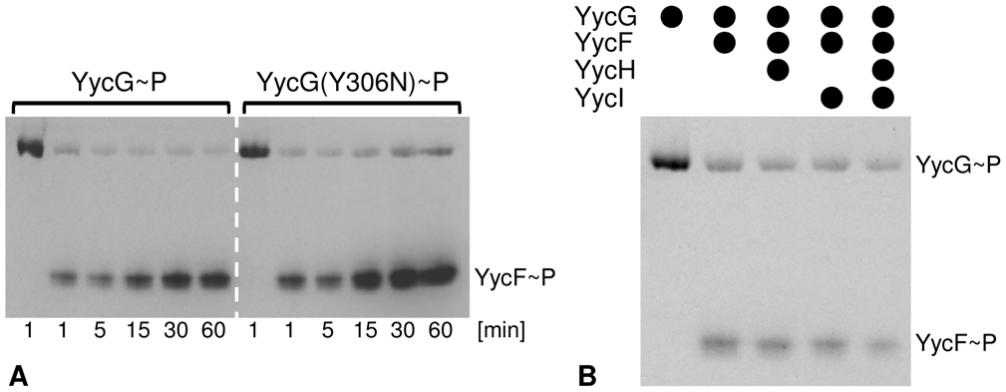
Phosphoryl group transfer to YycF in the detergent-micelle-model. (A) The phosphorylation activities of both kinase proteins in presence of their cognate response regulator YycF were observed in dependence of time. All phosphorylation assays were performed in presence of 8.5 mM Triton X-100, 25 µM ATP, 4 µCi [γ-^33^P]ATP and 500 mM KCl at RT. As a control the autophosphorylation activity of the YycG and the YycG(Y306N)-C-His_6_ kinase after 1 min incubation is shown. (B) Influence of YycH and YycI in the detergent-micelle-model. Phosphorylation activity of YycG in presence of various combinations of YycF, YycH and YycI is shown. After starting the reaction, the incubation was stopped for all samples after 60 s. All phosphorylation assays were performed in presence of 8.5 mM Triton X-100, 25 µM ATP, 4 µCi [γ-^33^P]ATP and 500 mM KCl at RT.

**Fig. 5 (A)** shows the phosphoryl group transfer by the full-length YycG kinases to its response regulator YycF employing 1.5 µg of each protein per assay. The phosphorylated form of YycF appeared as early as after 1 min on the autoradiography and after 30 min, a maximum signal intensity of YycF was reached, demonstrating that the kinase was able to phosphorylate its cognate response regulator in the detergent-micelle-model. In contrast to systems where a truncated ‘YycG had been tested [11], [17], the intensity of the phosphorylated band of the full-length YycG was rather weak in presence of the YycF-C-His_6_ response regulator and did not increase over time. In conclusion, these results indicate either an increased efficiency of phosphate group transfer by the full-length enzyme compared to the truncated ‘YycG proteins or an autodephosphorylation activity of full-length YycG in presence of YycF that had not been previously observed.

In *in vivo* studies with *Bacillus subtilis yycI* and *yycH* deletion mutants, a higher activity of the YycFG system has been observed, indicating that YycH and YycI might modify the kinase activity of YycG [5], [25]. **Fig. 5 (B)** shows the autoradiography of phosphorylation assays including YycF in the presence of the auxiliary proteins YycI and YycH (1.5 µg of each protein). In the detergent-micelle-model, only a very weak inhibitory effect of YycH and YycI was visible. It is likely that the detergent-micelle-model did not provide an optimal environment for this interaction, especially since a close proximity of TM domains is a prerequisite for protein-protein interaction of the whole Yyc complex [25].

### *In vitro* phosphorylation assays in the phospholipid-liposome-model

The fact that reconstitution into a membrane is a prerequisite for kinase activity has been observed for a number of other membrane bound histidine kinases (KdpD, DcuS and MtrB) [35]–[37]. In order to establish a test system that mimics the membrane embedded state, which represents the *in vivo* state of the YycG kinase much better than the detergent-micelle-model, both purified full-length C-His_6_-tagged protein variants were reconstituted with their TM regions into unilamellar vesicles (ULVs) to form proteoliposomes. Defined ULVs were prepared from polar *E. coli* phospholipids by extrusion through a polycarbonate membrane (pore diameter 400 nm). After partial solubilization of the ULVs by stepwise addition of a 20% Triton X-100 solution to a concentration that just exceeded saturation, the kinase (50 µg of protein /1500 µg of lipid) was added. The detergent was then removed using Bio-Beads and the proteoliposomes were collected by ultracentrifugation and extruded once more before they were shock frozen and stored at −70°C. Prior to use, the proteoliposomes were repeatedly extruded through a polycarbonate filter in order to restore a defined diameter.

In a first step, the autophosphorylation of the YycG kinases in the membrane environment provided by the phospholipid-liposomes was tested and the experiment was started by addition of [γ-^33^P]ATP. Experiments in buffer containing only 200 mM KCl and different concentrations of MgCl_2_ were unsuccessful in spite of prolonged incubation (60 min) and excessive exposure of X-ray films.

**Figure 6:**
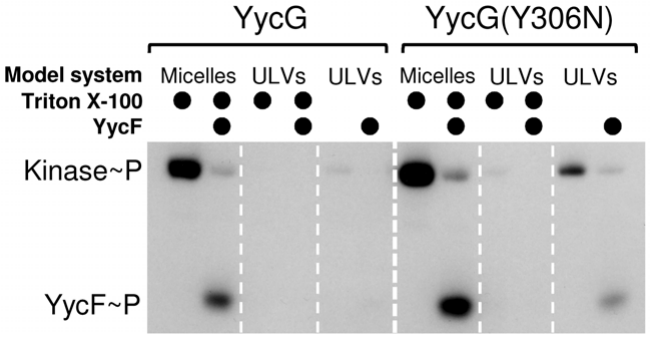
Comparison of the YycG-C-His_6_ and the YycG(Y306N)-C-His_6_ kinase activities. The experiments were performed in presence or absence of the cognate response regulator YycF in the detergent-micelle-model and the phospholipid-liposome-model, respectively. The activities of the kinases within the same model system can be compared directly. In the detergent-micelle-model 1.5 µg of the His-tag constructs of YycG, YycG(Y306N) and YycF were employed. In the phospholipid-liposome-model, equal amounts of YycG-C-His_6_ and YycG(Y306N)-C-His_6_ ULVs as determined by the concentration of the inorganic phosphate (in triplicate) were used and 1.5 µg of YycF-C-His_6_ were added to the ULVs. All phosphorylation reactions were started by the addition of 4 µCi [γ-^33^P]ATP and stopped after 60 min.

As in the detergent-micelle-model, an autophosphorylation of YycG was only visible when the buffer contained 500 mM KCl. The appearance of phosphorylated protein bands indicated that at least a part of the reconstituted kinases were inside-out orientated, i.e. the intracellular C-terminus with the ATPase domain was accessible for the [γ-^33^P]ATP on the surface of the liposomes. In the detergent-micelle-model both proteins were able to phosphorylate their cognate response regulator YycF-C-His_6_ after autophosphorylation, but YycG-C-His_6_ showed only a comparatively weak activity in the phospholipid-liposome-model, yielding rather weak bands in autoradiography (**Fig. 6**). This result might indicate that, in absence of a specific signal, the isolated wild type kinase is locked in a relatively inactive state in the membrane environment. In contrast, the mutant kinase YycG(Y306N)-C-His_6_ was characterized by a better activity in the liposome system.

**Figure 7:**
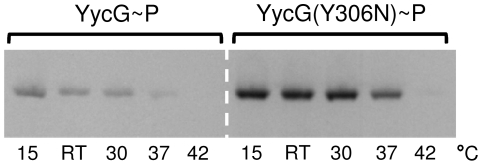
Influence of temperature/fluidity in the phospholipid-liposome-model. Equal amounts of YycG-C-His_6_ and YycG(Y306N)-C-His_6_ ULVs as determined by the concentration of the inorganic phosphate (in triplicate) were incubated at different temperatures to observe changes in activity due to changes in microviscosity of the ULVs. All phosphorylation reactions were started by the addition of 4 µCi [γ-^33^P]ATP and stopped after 60 min.

Since we assumed from the results with the Triton X-100 micelles, that the fluidity of the microenvironment might have a direct influence on kinase activity, this was also tested for the membrane embedded state of the kinase after reconstitution into phospholipid-liposomes (ULVs). Again, the variation of the temperature was used to cause changes in microviscosity of the proteoliposomes. **Fig. 7** shows that a decrease of activity can be observed for both kinases at temperatures above 37°C. In comparison, this effect is less pronounced than observed for the Triton X-100 micelles, but a correlation between the fluidity of the microenvironment and the activity of the kinase seems to be given. This assumption is underlined by the finding that the activities of both kinases were abolished by addition of Triton X-100 to the proteoliposomes, indicating that the partially solubilized proteins are not active if the fluidity is increased by addition of a surfactant (**Fig. 6**).

Furthermore, under standard conditions (RT and presence of 500 mM KCl) the transfer of the phosphoryl group to the response regulator was tested. YycF-C-His_6_ was added externally to the proteoliposomes and the reaction was again started by addition of [γ-^33^P]ATP and stopped after the desired incubation time by the addition of 5× SDS sample buffer. Identical amounts of soluble protein (1.5 µg) and equal amounts of proteoliposomes were employed for all assays in **Fig. 6**, which shows the activities of both kinases (YycG vs. YycG(Y306N)) and both systems (detergent-micelle-model and phospholipid-liposome-model) in comparison.

Since only little information could be gained about the interaction of the YycG kinase with the auxiliary YycH and YycI proteins in the detergent-micelle-model, reconstitution of all three proteins together into the membrane of ULVs was tested, but failed in the case of YycH (data not shown).

## Discussion

This paper represents for the first time the recombinant expression, purification and activity testing of the full-length YycG histidine kinase of *S. aureus*. Moreover, suitable *in vitro* conditions which enable demonstration of the phosphorylation activities of the full-length YycG kinase, employing detergent-micelles or functional reconstitution into phospholipid liposomes (ULVs), have been identified. Presumably, because of the experimental difficulties that had to be overcome in order to obtain good expression of the full-length protein in *E. coli* and to observe *in vitro* activity, several previous reports describe tests of the isolated cytoplasmic domains of YycG of *S. aureus* [11], [17], *S. epidermidis* [38], *B. subtilis* [39], [40] and *S. pneumoniae* [18]. The results presented here show that the conditions for the *in vitro* activity of the full-length kinase are clearly different from those of the truncated proteins: the isolated cytoplasmic histidine kinase domain (aa 220 to 609) of the truncated *S. aureus* ‘YycG protein showed good *in vitro* activity in phosphorylation buffers containing KCl concentrations of 200 mM [11] and the isolated kinase domain (truncated at aa 367) required the presence of ammonium ions for optimal activity (100 mM KCl and 100 mM NH_4_^+^) [17]. In contrast, the full-length YycG kinases of *S. aureus* were inactive in these buffers, also after reconstitution into detergent-micelles and phospholipid-liposomes (ULVs), and only the addition of elevated concentrations of alkali salts activated the enzymes. The strongest effects were observed with K^+^ and Rb^+^ and might be due to a change in the hydration state of the Triton X-100 micelles, which is accompanied by a change in microviscosity [29]–[33]. A variation of the temperature induced similar fluidity changes [31], [34] and a dramatic decrease in activity of both kinase variants at temperatures above RT supported the idea that YycG actually might respond to changes in fluidity of its microenvironment. A decrease in activity was also observed in the phospholipid-liposome-model. However, the effect of temperature was much less pronounced and appeared only at 37°C and higher temperatures. This finding may be explained by the fact that a biological membrane composed of phospholipids is more complex than a membrane mimicking surfactant like Triton X-100. In principle, the alkali cations bind to the carbonyl region of the membrane [41], however, the interactions between the ions and the bacterial membrane are influenced by its phospholipid composition. In pure PE membranes, the zwitterionic PE is densely packed, and therefore only a weak binding of sodium and hardly any interaction with potassium ions was observed [41]. In contrast, PG is an anionic lipid and in a mixture with PE, the PG promotes a strong formation of ion-lipid clusters and of hydrogen bonds between PE and PG and therefore leads to a more stable, compact and less dynamic bilayer [42]. The fact that we employed a polar lipid extract of *E. coli*, that contains PE and PG, to construct ULVs for reconstitution of YycG and that it was necessary to perform activity testing in presence of elevated KCl concentrations, could explain why the temperature showed a significantly lower effect in the intrinsically more stable phospholipid-liposome-model than in the detergent-micelle-model.

It was surprising, that after reconstitution into ULVs, only the mutant kinase showed high activity, whereas the wild type kinase seemed comparatively inactive, indicating that a further signal is still missing. Interestingly, in non-growing cells of *B. subtilis*, the activity of the YycG kinase is blocked by the binding of the two negative regulators YycH and YycI and, in the absence of a septum, the YycGHI complex is localized in the peripheral cell wall [6], [7], [23]–[25]. When the cell is growing, YycG co-localizes with the cell wall biosynthetic complex at the nascent septum, whereas the auxiliary YycH and YycI remain in the peripheral wall [23], [24]. The cytoplasmic PAS domain of YycG plays a crucial role for the localization of the kinase to the division site and might be acting as a cell-sorting module by interacting with proteins of the divisome (DivIB, Pbp2B, FtsL and FtsW) [23]. Here we found, that the mutant YycG(Y306N) kinase was more active than the wild type enzyme after reconstitution into phospholipid-liposomes (ULVs). Therefore, the exchange in the cytoplasmic PAS domain of YycG(Y306N)-C-His_6_ might override the intra-molecular signal transduction and activate the kinase in the absence of the proteins of the divisome.

**Figure 8:**
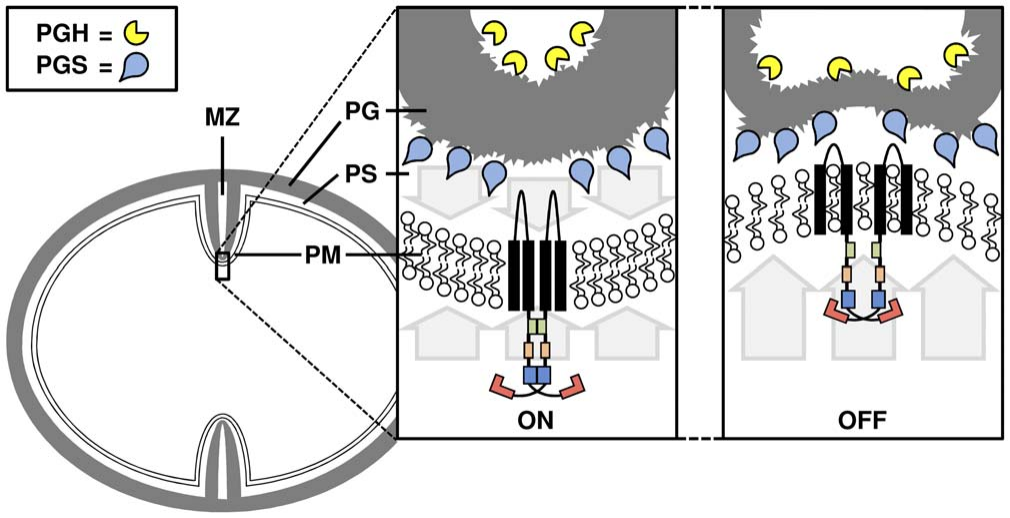
Model for the control of the YycG kinase activity by the membrane fluidity in its microenvironment. The organization of the growing septum in *S. aureus* and the localization of enzymes involved in peptidoglycan degradation and synthesis are depicted according to [44] who showed by cryo transmission electron micrography that the murein in the center of the nascent cell wall is hydrolyzed already during biosynthesis of the septum. Normally, the peptidoglycan (PG) ensures structural integrity of the cell by maintaining a counter pressure to the intracellular turgor which is symbolized by arrows. The turgor pressure of the cytoplasm and the counter pressure of the peptidoglycan also affect the tension, fluidity and stability of the plasma membrane (PM). A local weakness of the PG caused e.g. by an excess of degradation by peptidoglycan hydrolases (PGH) would also affect the state of the membrane and might be recognized via the TM domains of YycG, leading to a decrease of kinase activity and a concomitant decrease of the expression of the autolysin genes.

The main function of YycG in *S. aureus* seems to be coordination of cell wall homeostasis and turn-over, which is mainly regulated by cell wall degrading enzymes (autolysins) [4], [8], [23], [24], [43]. In the division septum, the kinase is assumed to be in an active mode and to stimulate transcription of the autolysin genes. In this context, an increase in membrane fluidity might function as a stop signal for YycG activity (**Fig. 8**). If the hydrolysis of the peptidoglycan in the center of the growing septum [44] proceeds beyond a crucial point that weakens the growing cell wall, the peptidoglycan and the cytoplasmic membrane should be distorted by the intracellular osmotic pressure. Such a bulge would increase the distance between the membrane lipids and cause local increases in fluidity. Exactly these microenvironmental changes might be sensed and transduced by the TM domains of YycG, thus inhibiting the activity of the kinase. A related mechanism for the sensing of temperature induced changes in membrane thickness has been recently elucidated for DesK of *B. subtilis* [45].

For the *B. subtilis* YycFGHI complex an octameric model for the Yyc proteins interacting via their transmembrane domains (H↔I↔G_2_↔G_2_′↔I′↔H′) has been proposed [25]. This octameric model is compatible with several reports suggesting that the auxiliary proteins YycH and YycI, beyond their function in controlling the activity of YycG kinase, might also play a role in sensing, processing and maybe in transduction of further signals that regulate the YycFGHI system [5], [6], [21], [23]. The observed oligomeric organization (up to hexamers) of the auxiliary YycI protein of *S. aureus* during BN-Page in this work might indicate that the composition of the suggested octameric complex could be much more dynamic than expected, i.e. the different oligomeric states of YycI might interfere with the indirect interaction between YycH and YycG and thereby fine-tune kinase activity. In contrast to a previous suggestion [22], we were not able to confirm dimerisation of YycH.

In summary, we have shown here that the full-length kinase YycG of *S. aureus* can be expressed in *E. coli* and can be tested in a detergent-micelle-model as well as after reconstitution in ULVs (phospholipid-liposome-model). In the presence of alkali salts or at different reaction temperatures, respectively, the full-length YycG kinase alters its activity in response to changes in its microenvironment, which are most likely represented by fluctuations in membrane fluidity. Therefore, especially the phospholipid-liposome-model will be the foundation for further studies and will hopefully expand the understanding of this fascinating regulatory system in *S. aureus* as well as in other bacteria.

## Materials and Methods

### Construction of the *E. coli* expression host strains harbouring pREP4groESL(MT)

To avoid formation of inclusion bodies and to increase the amount of soluble recombinant protein, we co-expressed the *E. coli* chaperone proteins GroES and GroEL. In contrast to Amrein and colleagues [46], *groESL* expression was not inducible, but constitutive in our system. To this end, a fragment comprising *groESL* of *E. coli* JM109 was amplified by PCR using the oligonucleotides listed in **Table 1**. The forward primer harboured a suitable ribosome binding site (AGGAGA) downstream of the restriction site. The resulting PCR fragment and the plasmid pREP4 (Qiagen) were digested with *Nhe*I and *Sal*I and ligated, generating pREP4groESL(MT). After transformation of *E. coli* JM109 with the ligation mixture, the recombinant plasmid was shuttled into the *E. coli* strains BL21(DE3) (Novagen), C41(DE3) or C43(DE3) [19] to create the expression host strains *E. coli* BL21(DE3) pREP4groESL(MT), *E. coli* C41(DE3) pREP4groESL(MT) and *E. coli* C43(DE3) pREP4groESL(MT). Plasmid integrity was ensured by sequencing (Sequiserve, Vaterstetten, Germany).

### Cloning, overexpression and purification of YycF (VicR), YycG (VicK), YycH and YycI

The *yycF* (*vicR*), *yycG* (*vicK*), *yycH* and *yycI* genes of *S. aureus* strain 137/93A [47] were amplified using the oligonucleotides listed in **Table 1**. To express the mutant version of the YycG kinase that harbours a non-silent nucleotide exchange (T916A) leading to a point mutation in the putative signal sensing PAS domain, the plasmid pTvicRK(Y306N) [14] was used as a template. The *Nco*I restriction site engineered in all forward primers provided an alternative start codon. To preserve the original start codon of all genes and to avoid a frame shift during translation, the restriction site *Nco*I was placed in frame, yielding recombinant Yyc proteins that possess two additional residues (Met-Gly) at their N-termini. The resulting PCR fragments were digested with *Nco*I and *Xho*I and cloned into the corresponding sites of the vector pET22bΔpelB [20], generating pET22bΔpelBvicR, pET22bΔpelBvicK, pET22bΔpelByycH and pET22bΔpelByycI. Plasmid integrity was ensured by sequencing (Sequiserve, Vaterstetten, Germany). All plasmids were subcloned in the *E. coli* host strain JM109. For overexpression of C-terminally His_6_-tagged Yyc proteins encoded on the pET22bΔpelB-derived plasmids, the recombinant vectors were transformed into the *E. coli* strains BL21(DE3) pREP4groESL(MT), C41(DE3) pREP4groESL(MT) or C43(DE3) pREP4groESL(MT). Expression cultures were grown in 0.5 l or 1.0 l of lysogeny broth (LB) containing ampicillin (40 mg/l) and kanamycin (25 mg/l) to ensure plasmid stability of the cells. Cultures were incubated with shaking at 30°C to an optical density at 600 nm (OD_600_) of 0.5–0.6. Then protein expression was induced by addition of isopropyl-β-D-thiogalactopyranoside (IPTG) to a final concentration of 1 mM and incubation of the expression cultures was continued for 4 h or 16 h (over night) under the same conditions. Cells were pelleted by centrifugation at 6,800× *g* for 6 min and resuspended in 15 to 20 ml of ice cold buffer L (50 mM NaH_2_PO_4_, 300 mM NaCl, 10 mM imidazole at pH 8) and stored at −20°C until further processing. To purify the Yyc proteins, the cells were disrupted by sonication in presence of lysozyme (1 g/l). All subsequent steps were performed at 4°C. After disruption, RNase A (10 mg/l) and DNase I (5 mg/l) were added and cell debris was removed by centrifugation (15,000× *g* for 10 min). One millilitre of Ni-NTA-Agarose (Qiagen, Hilden, Germany) was added to the crude protein supernatant. After stirring for 2 h, the suspension was poured into a column equilibrated with 3 ml of buffer L. The column was washed twice with 4 ml of buffer W1 (50 mM NaH_2_PO_4_, 300 mM NaCl, 20 mM imidazole at pH 8) and 4 ml of buffer W2 (50 mM NaH_2_PO_4_, 300 mM NaCl, 40 mM imidazole at pH 8). The Yyc proteins were eluted with an imidazole gradient of 40 to 300 mM. In the case of membrane bound proteins (i.e. both YycG kinases and YycI), the supernatant obtained after sonication of the cells and centrifugation was additionally ultracentrifuged at 218,000× *g* for 60 min at 4°C to harvest the membrane fraction which was used for purification. Furthermore, all buffers employed for purification of these proteins additionally contained 2 mM β-mercaptoethanol, 30% (v/v) glycerol and up to 39 mM n-dodecyl-β-d-maltoside (DDM). After elution, pure glycerol was added to a final concentration of 50% (v/v) and all protein fractions were stored at −20°C until further processing. To remove imidazole and detergent and to concentrate the protein solution, the fractions were pooled and dialyzed against dialysis buffer I (50 mM NaH_2_PO_4_, 300 mM NaCl, 50% (v/v) glycerol at pH 8) or dialysis buffer II (50 mM HEPES, 200 mM KCl, 50% (v/v) glycerol at pH 8) utilizing Slide-A-Lyzer dialysis cassettes with a molecular weight cut-off of 10 kDa and 0.1–0.5 ml capacity (Thermo Scientific, Bonn, Germany). Determinations of the protein concentration were carried out according to Bradford [48].

### Blue native polyacrylamide gel electrophoresis (BN-PAGE) and oligomeric state analysis

To analyze the oligomeric states of the Yyc proteins, BN-PAGE was performed as previously described [26] with some modifications. Five µg of YycF, YycH and YycI, and 10 µg of YycG and YycG(Y306N), respectively, in 10 µl of dialysis buffer I were mixed with 5 µl 0.5% (w/v) Coomassie Brilliant Blue R250 and loaded onto a 4–20% Tris-HCl precast native gradient gel (Bio-Rad, Munich, Germany). The BN-PAGE was performed at a constant voltage of 80 V and cooling (4–7°C) for 3–4 h in buffer BN (25 mM Tris, 192 mM glycine, 0.02% (w/v) Coomassie Brilliant Blue R250 at pH 8.5). The protein bands were analyzed after incubation at RT in destaining solution (45% (v/v) methanol and 10% (v/v) acetic acid) to remove excess Coomassie Blue. For all C-His_6_-tagged constructs theoretical protein parameters, like pI values and molecular weight on basis of the amino acid sequence, were determined by utilization of the ExPASy online tool “ProtParam” (free access under http://web.expasy.org/protparam/).

### Construction of liposomes and proteoliposomes

Protein reconstitution into liposomes was performed according to the principles described by Rigaud and Levy [49]. These methods previously were successfully adapted by Möker et al. [37] for proteoliposome construction with MtrB and in the following the whole procedure with further modifications for a functional reconstitution of both purified YycG-C-His_6_ kinases is described. To prepare a defined lot of lipid cakes, lyophilized *E. coli* polar lipid extract (Avanti Polar Lipids, Alabaster, AL) was dissolved in chloroform/methanol (1∶1) to a final concentration of 20 g/l. 70 µl aliquots of the solution (1.5 mg) were pipetted into reaction tubes and the solvent was removed by evaporation and centrifugation under vacuum. After reaching dryness, the reaction tubes were sealed under a nitrogen atmosphere to avoid oxidation, and stored at −20°C until further processing. Prior to reconstitution, a lipid cake aliquot was dissolved in a total volume of 1 ml extrusion buffer (100 mM potassium phosphate buffer, pH 7.5) to form undefined large multilamellar vesicles (LMVs). To obtain defined unilamellar vesicles (ULVs), the LMV suspension was extruded (Mini-Extruder, Avanti Polar Lipids, Alabaster, AL) 19 times through a polycarbonate filter (pore diameter 400 nm). Before reconstitution, the liposomes were solubilized by stepwise addition of Triton X-100 (20% in extrusion buffer). The insertion of detergent was followed by measurement of the turbidity of the solution at 540 nm. After saturation with detergent had been reached, the liposomes were incubated for 20 min at RT under gentle stirring and subsequently mixed with the purified YycG-C-His_6_ or YycG(Y306N)-C-His_6_ kinase in a ratio of 1∶31 (protein to lipid (w/w)). The mixture was incubated for additional 35 min at RT under gentle stirring. To remove the detergent, an aliquot of Bio-Beads SM-2 adsorbent (Bio-Rad, Munich, Germany), pre-washed with ultra-pure water, was added in a ratio of 5∶1 (Bio-Beads (wet weight) to Triton X-100). The mixture was gently stirred at RT for 1 h, then, another aliquot of fresh Bio-Beads was added. After another hour of gentle stirring, two more aliquots of fresh Bio-Beads were added, and the mixture was stirred at 4°C. After incubation for 16 h over night, a final aliquot of fresh Bio-Beads was added and the mixture was gently stirred for 1 h at 4°C. After sedimentation of the Bio-Beads, the supernatant was ultracentrifuged at 414,000× *g* for 25 min at 25°C. The supernatant was discarded and the proteoliposomes were resuspended in 1 ml of 1×CL10 buffer (50 mM HEPES, 500 mM KCl, 0.1 mM EDTA, 5 mM MgCl_2_, 0.5 mM DTT, 10% (v/v) glycerol at pH 8), extruded 19 times through a polycarbonate filter (pore diameter 400 nm), ultracentrifuged at 649,000× *g* for 25 min at 25°C, and resuspended in 155 µl 1×CL10 buffer. Aliquots of 50 µl were quickly frozen in liquid nitrogen and stored at −70°C until further use.

### Phosphorylation assays

For the first phosphorylation studies in the detergent micelle system, 1.5 µg of the native or mutated YycG kinase were incubated in the absence or in the presence of their cognate response regulator YycF in 1× CL3.5 phosphorylation buffer (50 mM HEPES, 500 mM KCl, 0.1 mM EDTA, 5 mM MgCl_2_, 0.5 mM DTT, 3.5% (v/v) glycerol at pH 8) with 8.5 mM Triton X-100. For tests with further alkali salts in different concentrations the buffer has been modified as indicated. To initiate the phoshorylation reaction in a final volume of 10 µl, a mix of “cold” and “hot” ATP was added to a final concentration of 25 µM ATP and 4 µCi [γ-^33^P]ATP. After starting the reaction, the samples were incubated at RT or as indicated and stopped at desired time points by the addition of 6 µl of 5× SDS sample buffer. All samples were loaded directly - without heating - onto a 10% Bis-Tris precast gel (Invitrogen, Karlsruhe, Germany) and analyzed by SDS-PAGE in presence of MOPS SDS running buffer (Invitrogen) according to the manufacturers' instructions. The presence of an antioxidant and reducing agent during SDS-PAGE as recommended by the manufacturer (Invitrogen) did not lead to better results and was therefore omitted. SDS-PAGE was followed by autoradiography.

After reconstitution of the YycG kinase into ULVs to form proteoliposomes (see above), a previously prepared 50 µl proteoliposome aliquot was resuspended in 950 µl 1× CL3.5 phosphorylation buffer and freshly extruded (Mini-Extruder, Avanti Polar Lipids) for 19 times through a polycarbonate filter (pore diameter 400 nm) and ultracentrifuged at 649,000× *g* for 25 min at 25°C prior to use in the phosphorylation assays. After discarding the supernatant, the proteoliposomes were resuspended in 30–50 µl of 1× CL3.5 phosphorylation buffer. To enable a direct comparison of the activities of YycG-C-His_6_ and YycG(Y306N)-C-His_6_ in the phospholipid-liposome-model, the concentration of inorganic phosphate (P_i_) was determined according to Rouser [50] in triplicate as a measure of the concentration of proteoliposomes. Proteoliposomes with the native or mutated YycG protein were incubated in the absence or in the presence of 1.5 µg of externally added YycF in 1× CL3.5 phosphorylation buffer. To initiate the phosphorylation reaction, 4 µCi [γ-^33^P]ATP were added to a final test volume of 10 µl and the samples were incubated at RT or as indicated and stopped after 60 min by the addition of 5× SDS sample buffer. The analysis was performed by SDS-PAGE and autoradiography as described above.
